# Reference Ranges of Left Ventricular Hemodynamic Forces in Healthy Adults: A Speckle-Tracking Echocardiographic Study

**DOI:** 10.3390/jcm10245937

**Published:** 2021-12-17

**Authors:** Francesco Ferrara, Francesco Capuano, Rosangela Cocchia, Brigida Ranieri, Carla Contaldi, Graziella Lacava, Valentina Capone, Salvatore Chianese, Salvatore Rega, Roberto Annunziata, Chiara Sepe, Andrea Salzano, Rodolfo Citro, Antonello D’Andrea, Ciro Mauro, Filippo Cademartiri, Gianni Pedrizzetti, Eduardo Bossone

**Affiliations:** 1 Cardio-Thoracic-Vascular Department, University Hospital “San Giovanni di Dio e Ruggi d’Aragona”, 84125 Salerno, Italy; fferrara1975@gmail.com (F.F.); rodolfocitro@gmail.com (R.C.); 2 Department of Mechanics, Mathematics and Management, Polytechnic University of Bari, 70126 Bari, Italy; francesco.capuano@poliba.it; 3 Cardiology Division, A Cardarelli Hospital, 80131 Naples, Italy; rosangela.cocchia@aocardarelli.it (R.C.); caponevalentina92@libero.it (V.C.); sasichian@gmail.com (S.C.); dr.robertoannunziata@gmail.com (R.A.); chiara.sepe@aocardarelli.it (C.S.); ciro.mauro1957@gmail.com (C.M.); 4 IRCCS SDN, 80143 Naples, Italy; brigida.ranieri@synlab.it (B.R.); andre.salzano@gmail.com (A.S.); filippocademartiri@gmail.com (F.C.); 5 Heart Failure and Rehabilitative Cardiology Unit, AO dei Colli, Monaldi Hospital, 80131 Naples, Italy; contaldi.carla@gmail.com; 6 Anesthesia and Intensive Care, University Hospital “San Giovanni di Dio e Ruggi d’Aragona”, 84125 Salerno, Italy; graziellalacava1979@libero.it; 7 Department of Translational Medical Sciences, Federico II University, 80131 Naples, Italy; salreg25@gmail.com; 8 Unit of Cardiology, Department of Traslational Medical Sciences, University of Campania “Luigi Vanvitelli”, Monaldi Hospital, 80131 Naples, Italy; antonellodandrea@libero.it; 9 Department of Engineering and Architecture, University of Trieste, 34127 Trieste, Italy; giannip@dia.units.it

**Keywords:** speckle-tracking echocardiography, hemodynamic forces, intraventricular pressure gradient, left ventricle, strain

## Abstract

Background: The normal limits of left ventricular (LV) hemodynamic forces (HDFs) are not exactly known. The aim of this study was to explore the full spectrum of HDF parameters in healthy subjects and determine their physiologic correlates. Methods: 269 healthy subjects were enrolled (mean age: 43 ± 14 years; 123 (45.7%) men). All participants underwent an echo-Doppler examination. Tri-plane tissue tracking from apical views was used to measure 2D global endocardial longitudinal strain (GLS), circumferential strain (GCS), and LV HDFs. HDFs were normalized with LV volume and divided by specific weight. Results: LV systolic longitudinal HDFs (%) were higher in men (20.8 ± 6.5 vs. 18.9 ± 5.6, *p* = 0.009; 22.0 ± 6.7 vs. 19.8 ± 5.6, *p* = 0.004, respectively). There was a significant correlation between GCS (increased) (r = −0.240, *p* < 0.001) and LV longitudinal HDFs (reduced) (r = −0.155, *p* = 0.01) with age. In a multivariable analysis age, BSA, pulse pressure, heart rate and GCS were the only independent variables associated with LV HDFs (β coefficient = −0.232, *p* < 0.001; 0.149, *p* = 0.003; 0.186, *p* < 0.001; 0.396, *p* < 0.001; −0.328, *p* < 0.001; respectively). Conclusion: We report on the physiologic range of LV HDFs. Knowledge of reference values of HDFs may prompt their implementation into clinical routine and allow a more comprehensive assessment of the LV function.

## 1. Introduction

Left ventricular (LV) function is a major diagnostic and prognostic determinant of a wide range of cardiac diseases [1,2,3]. The LV ejection fraction (EF) is the most commonly used parameter to evaluate LV function, although it only provides global information about the chamber contraction. In this regard, the development and clinical implementation of speckle-tracking echocardiography (STE) have allowed the study of the regional myocardial deformation by global longitudinal strain (GLS) and global circumferential strain (GCS) indices to be more effective in detecting subclinical alteration of LV function compared to LV EF [4,5,6,7,8,9]. Furthermore, advances in post-processing of standard STE technology have permitted to estimate the intraventricular pressure gradients (IVPG), that drive blood flow during LV ejection and filling [10,11,12]. This has led to the definition of the LV hemodynamic forces (HDFs) as IVPG averaged over the LV volume, reflecting the forces effectively exchanged between the blood flow and the LV walls [13,14]. HDFs were proposed as an indicator of the correct sequence of IVPGs generation and thus of LV pumping function [15]. Recent studies have demonstrated that parameters based on HDF compared to LV EF and strain may provide additional information about structural and functional LV adaptations, representing promising markers for the identification of silent sub-clinical myocardial dysfunction [16,17,18,19,20]. Interestingly, innovative mathematical developments demonstrated that the HDFs in the LV can be computed exactly through a proper post-processing of results of standard STE [21,22]. However, the normal limits and physiologic correlates of LV HDFs have not been fully investigated [20,21,22]. Therefore, the aim of this study was to explore the full range of LV HDFs indices along with clinical and echocardiographic correlates in a large cohort of healthy subjects.

## 2. Methods

### 2.1. Study Population

The study population consisted of 269 healthy subjects (volunteers or subjects referred for work ability assessment; mean age 43.4 ± 14.0; 123 (45.7%) men) [23,24]. They underwent, at the echocardiographic laboratory of the Cardiology Division, “Cava de’Tirreni-Amalfi Coast”, Heart Department, University Hospital of Salerno, full screening for cardiovascular disease, including a questionnaire on medical history, use of medications, cardiovascular risk factors and lifestyle habits (alcohol intake, smoking, physical activity) [23,24]. Physical examinations (height, weight, heart rate (HR) and blood pressure (BP)) and clinical assessments were conducted according to standardized protocols by trained and certified staff [23,24]. Body surface area (BSA) was calculated according to the DuBois formula (0.20247 × height (m) 0.725 × weight (kg) 0.425). Three BP measurements were obtained from the right arm by sphygmomanometer and the results were averaged to determine systolic and diastolic BP. Pulse pressure (PP) was calculated as systolic BP (SBP)–diastolic BP (DBP). The study was approved by the institution’s ethics board and informed consent was obtained from all participants [23,24]. 

### 2.2. Conventional Echocardiography

Transthoracic echocardiography (TTE) examinations were performed with commercially available equipment on all subjects (Vivid E9—GE Healthcare, Milwaukee, WI, USA). Two independent experienced operators (FF, RC), blinded to the clinical data, performed offline left and right heart measurements and speckle-tracking (ST) analysis according to the current recommendations of the American Society of Echocardiography/European Association of Cardiovascular Imaging [25,26,27]. Specific views included the parasternal long- and short-axis views, apical 4, 2 and 3 chamber views and subcostal views, including respiratory collapse of the inferior vena cava (IVC). Pulsed and continuous wave Doppler interrogation was performed on all 4 cardiac valves. Specific average measurements were taken of the 5 cardiac cycles. M-mode measurements were performed in the parasternal long-axis view with the patient in the left lateral position and included left ventricular internal diameter in diastole (LVIDd) and systole (LVIDs), the interventricular septum in diastole (IVSDd) and the inferolateral wall in diastole (ILWTDd). LV mass was calculated by the Penn convention and indexed for BSA [25]. The LV EF was calculated from LV end-diastolic and end-systolic volumes by modified Simpson’s equation in the apical 4 and 2 chamber views [25]. Valvular regurgitation was quantified from color Doppler imaging and categorized as absent, minimal (within normal limits), mild, moderate or severe [25]. Doppler-derived LV diastolic inflow was recorded in the apical 4-chamber view by placing the sample volume at the tip of the mitral valve leaflets. The following LV diastolic parameters were measured: E and A peak velocities (m/s) and their ratio and E wave deceleration time (ms). The early (e’) diastolic velocities were measured by tissue Doppler imaging (TDI) at the septal and lateral corner of the mitral annulus and the mean between the two values was calculated. Mitral E velocity, corrected for the influence of relaxation (i.e., the E/mean e’ ratio), was assessed to estimate LV filling pressures [26]. LV stroke volume (SV) was calculated as the product of LV outflow tract area and outflow tract velocity time integral (VTI). Cardiac output (CO) was calculated using the following formula: CO (L/min) = HR × LV SV [23,24,25]. 

### 2.3. Speckle-Tracking Analysis

**Figure 1:**
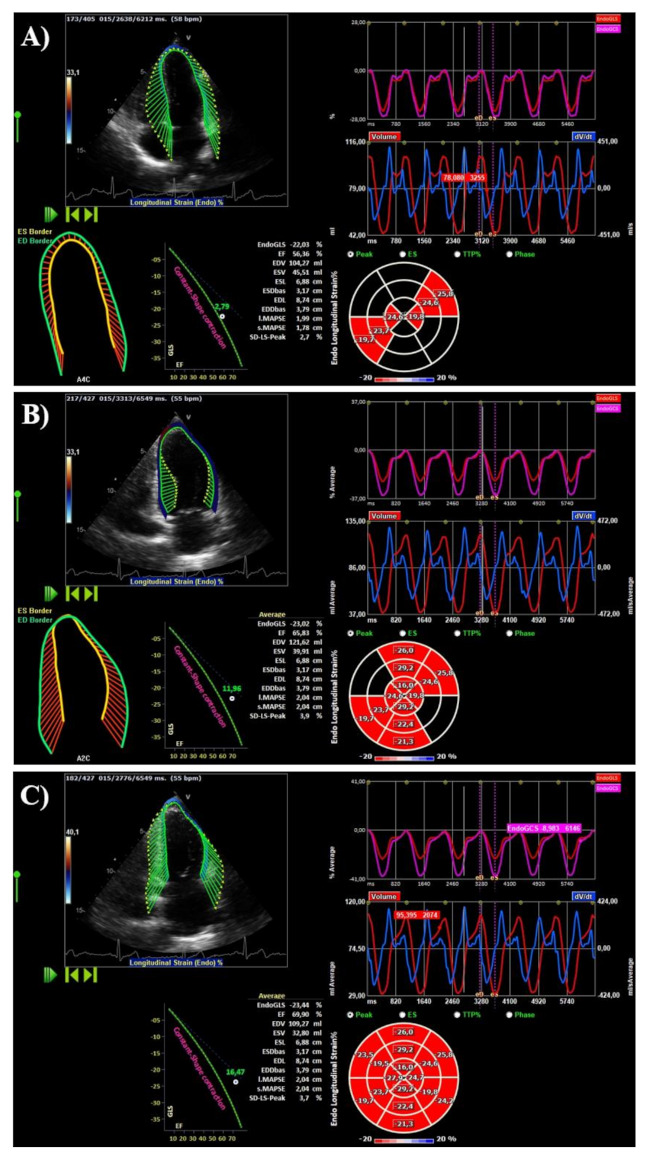
Post processing steps. (**A**) Analysis of apical 4 chamber view with vector velocity imaging, representation of endo GCS and GLS, variation of volumes and dV/dt, segmental longitudinal strain showed as bull-eye. (**B**) Analysis of 2 chamber view. (**C**) Analysis of 3 chamber view with average data. GCS, global circumferential strain; GLS, global longitudinal strain.

We included healthy subjects with frame rate acquisition of at least 50 Hz and with optimal images of all 18 segments in order to conduct strain-based HDFs analysis. In this regard, 37 subjects were excluded due to poor imaging quality (total study population = 269). 2D images from apical views (4-, 3- and 2-chamber) were re-analyzed offline using a commercially available software (2D-CPA v.1.4; TomTec Imaging Systems Gmbh, Unterschleissheim, Germany). The cardiac cycle was selected where the endocardial borders were better visible both in diastole and in systole. The LV end-systolic endocardial borders in each apical view were drawn and the software tracked the endocardial borders over the entire heartbeat. When necessary, the end-diastolic borders were corrected manually with consequent automatic propagation of the border correction over the entire cycle to match the original end-systolic borders. The LV end-systolic and end-diastolic volumes were calculated according to the modified Simpson rule [4,25,28]. Speckle-tracking analysis was performed to obtain 2D global longitudinal strain (GLS) and global circumferential strain (GCS), including 6 segments in each of the 3 apical views (4-, 3- and 2-chamber). Segments in which no adequate tracking quality could not be obtained despite manual adjustment being excluded. The 2D GLS and GCS were calculated as the average of 18 myocardial segments recorded in the three apical views [4,28] (Figure 1). The end-systolic GLS and GCS was then extracted as the main deformation parameter. 

### 2.4. Hemodynamic Forces

HDFs were originally computed from the blood velocity in the LV volume [15,16,17]. A recent mathematical formulation, based on the balance of momentum, demonstrated that the same quantity can also be evaluated from the motion of the endocardium and by the average flow across the mitral valve and outlet tract [22]. The mitral diameter was evaluated by the average in the three images view at end-diastole, measuring the internal edge of the valve annulus: (a) parasternal long-axis view (anterior–posterior diameter); (b) apical four-chamber view; and (c) apical two-chamber view. The aortic diameter was measured at peak systole in the 3-chamber projection from the inner edge of the valve. This formulation was included in a prototype version of the previously mentioned software for STE; an analogous prototype was previously used with feature tracking in cine magnetic resonance imaging (MRI) [19].

**Figure 2:**
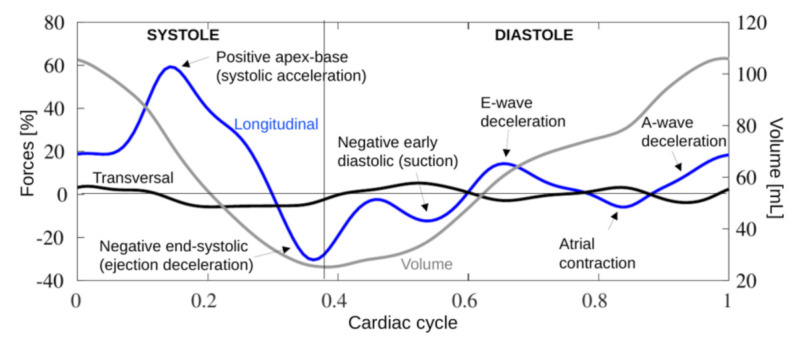
Typical time evolution of left ventricular hemodynamic forces over the cardiac cycle. The blue line shows apical-basal LV longitudinal forces, larger positive compared with transversal components (black line). Acceleration toward the base accounts for the systolic peak acceleration, after which deceleration causes the late systolic negative peak. Coinciding with the onset of diastole, another small negative early peak (suction) appeared, corresponding to early passive filling of the left ventricle (E-wave). A similar pattern was seen toward the end of diastole, corresponding to atrial contraction (A-wave). The grey line shows the LV volume over the cardiac cycle.

From the three endocardial borders estimated by speckle-tracking during the cardiac cycle, and from the knowledge of the aortic root and mitral inflow dimensions, the strain software also computed the time profile of the longitudinal (apex-base, HDF_L_) and transversal (inferolateral-anteroseptal, HDF_T_) components of HDFs. To improve comparability between different subjects, forces were expressed in dimensionless form after normalization with the LV volume and specific weight, and expressed as a percentage of gravity acceleration. A typical time evolution of the HDF components over the cardiac cycle is illustrated in Figure 2, superimposed with the LV volume. 

**Figure 3:**
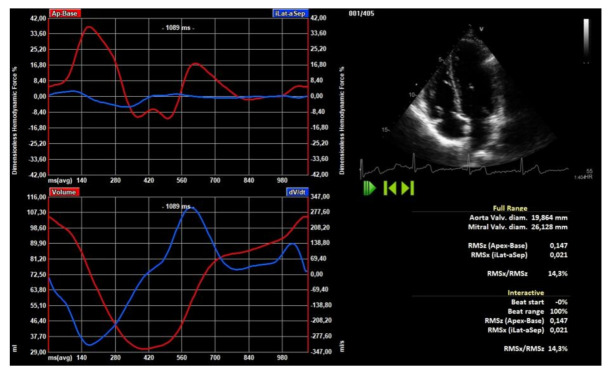
Representation of dimensionless hemodynamic forces obtained by speckle tracking analysis.

From the time-profile, some physically-based parameters were extracted for numerical comparisons. The HDF parameters were: (a) the systolic impulse, computed as the area under the curve of the longitudinal force during the positive interval of the systolic phase [29], this value was normalized with the time interval thus providing the systolic time average; (b) the systolic peak of the longitudinal force during the systolic phase; (c) the average amplitude, computed as the root mean square (rms) of both components (longitudinal and transversal) over the whole cardiac cycle; (d) the average amplitude during systole only and; (e) during diastole; (f) the alignment of the force with the LV axis, an angle ranging from 0° (perfect longitudinal alignment) to 90° (completely transversal) computed by the average angle during the cardiac cycle [7]. The calculation of the parameters of the LV HDF was performed using a commercially available software (2D-CPA v.1.4; TomTec Imaging Systems Gmbh, Unterschleissheim, Germany) (Figure 3).

### 2.5. Statistical Analysis

Continuous variables were expressed as means ± standard deviation (SD). Normal distribution of the continuous values was assessed by the Kolmogorov–Smirnov test. Differences between groups were analyzed by unpaired Student *t*-test. *p*-values less than 0.05 were considered as statistically significant. As lower and upper limits for normal HDFs, we used 95% confidence intervals (CI) and/or ± 2 SD. The Pearson’s correlation analysis was used to determine correlations between continuous variables. The variables were selected according to their clinical relevance and potential impact on LV HDFs. Multivariable linear regression analysis, including all significant clinical and echocardiographic parameters from the univariate analysis was constructed to assess the independent associations of these variables with LV HDFs. We reported the standardized beta coefficients in regression analysis. The inter- and intra-observer variabilities were examined using both paired t-tests and intraclass correlation coefficient (ICC) in 30 randomly selected cases. An ICC of >0.8 indicated good agreement. Data were processed using MATLAB^®^ (Mathworks, Natick, MA, USA).

## 3. Results

### 3.1. Demographic Data

The demographic data of the study population are reported in Table 1. A total of 123 men (mean age 43 ± 14 years) and 146 women (mean age 44 ± 14 years) were included. Compared with men, women had a lower height and weight, lower BSA and body mass index (BMI), lower BP and higher HR. 

### 3.2. Left Heart Echo-Doppler Analysis

Women had smaller LV wall thicknesses, LV dimensions, LV mass, left atrial volume and LV volume (all *p* < 0.001). On the other hand, compared to women, men had slightly higher LV ejection fraction (*p* = 0.049). No significant sex-related differences in mitral peak E/e’ ratio were noted (*p* = 0.319). However, men demonstrated higher SV (*p* < 0.001) and CO (*p* = 0.005) (Table 2).

### 3.3. Right Heart Echo-Doppler Analysis

Right heart dimensions were larger in men (all *p* < 0.001). No significant sex-related differences in tricuspid annular plane systolic excursion (TAPSE) (*p* = 0.916), S’ (*p* = 0.251) and pulmonary artery systolic pressure (*p* = 0.170) were found. Compared to women, men had slightly shorter right ventricular outflow tract (RVOT) acceleration time (Act) (*p* = 0.049) (Table 3).

### 3.4. LV Deformation

The values of end-systolic global LV strain for the overall population were GLS = −23.1 ± 1.5 and GCS = −33.0 ± 3.9. No significant sex-related differences of the mean values of GLS and GCS were found (*p* = not significant (ns)) (Table 4).

### 3.5. LV Hemodynamic Forces

The mean values of HDFs parameters of the overall population were: (a) systolic impulse = 19.8% ± 6.1, with (b) average amplitude of the longitudinal component in the entire heartbeat = 15.0% ± 4.4, that was higher during systole = 20.8% ± 6.2 and lower during diastole = 8.1% ± 2.7. LV systolic impulse (%) and LV systolic longitudinal force (%) were higher in men (20.8 ± 6.5 vs. 18.9 ± 5.6, *p* = 0.009; 22.0 ± 6.7 vs. 19.8 ± 5.6, *p* = 0.004, respectively). The lower limits of normal of the longitudinal force in the whole cycle were 6.3% in men and 6.5% in women. Systolic longitudinal force was 8.8% in both sexes. The amplitude values were lower for the transversal component. LV transversal force (%) during the whole cycle and systole was higher in men (2.6 ± 1.0 vs. 2.2 ± 0.8, *p* = 0.004; 2.9 ± 1.1 vs. 2.5 ± 0.9, *p* < 0.001, respectively). The lower limits of normal of the transversal HDFs in the whole cycle and during systole were 0.6% and 0.7% in both sexes, respectively. No significant sex-related differences during diastole for longitudinal and transversal components were noted (*p* value = ns). On average, the force showed a good alignment of 14° ± 3.6 with the LV axis, slightly greater in women (Table 5). 

### 3.6. Clinical and Echocardiographic Correlates of LV Hemodynamic Force

**Figure 4:**
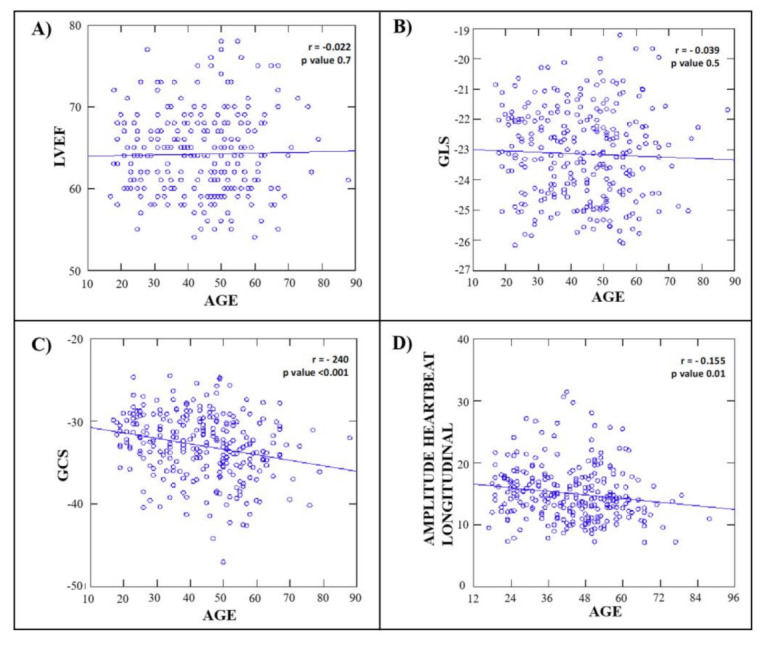
LVEF (**A**); GLS (**B**); GCS (**C**); amplitude heart beat longitudinal and (**D**) hemodynamic forces plotted against age in overall population. LVEF, left ventricular ejection fraction; GLS, global longitudinal strain; GCS, global circumferential strain.

No significant correlation between left ventricular ejection fraction (LVEF) and GLS with age was found (r = −0.022 and r = −0.039, respectively, all *p* = ns). GCS showed a statistically significant correlation with age (r = −0.240; *p* < 0.001) (Figure 4). 

In univariate analysis, LV longitudinal HDF was negatively correlated with age (r = −0.155, *p* = 0.01), BSA (r = 0.390; *p* = 0.02), pulse pressure (r = 0.206; *p* = 0.001) and heart rate (r = 0.480; *p* < 0.001). Furthermore, LV longitudinal force was weakly correlated with GLS (r = −0.153; *p* = 0.01) and more significantly with GCS (r = −0.254; *p* < 0.001). On the opposite, no significant correlation between LV longitudinal HDFs and LV mass, LA volume, LVEF (%), stroke volume and E/e’ was found (all *p* = ns) (Table 6). 

In a multivariable analysis age, BSA, pulse pressure, heart rate and GCS were the only independent variables associated with LV longitudinal HDFs (β coefficient = −0.232, *p* < 0.001; 0.149, *p* = 0.003; 0.186, *p* < 0.001; 0.396, *p* < 0.001; −0.328, *p* < 0.001, respectively) (Table 6). 

### 3.7. Inter and Intra-Observer Variability

The quality control process was designed to be simple, reproducible and sustainable. Intra-observer variability was tested in 2 observers (FF, RC), who volunteered to repeat the measurement session of images in 20 randomly selected cases on two separate days. The intra-observer quality control analysis revealed an excellent ICC of 0.98 for GLS and GCS (95% CI: 0.95 to 0.99) and ICC of 0.97 for HDF (95% CI: 0.95−0.99) (all *p* < 0.01) (Table 7).

Inter-observer variability was tested in two blinded and independent observers (FF, RC). Reproducibility analyses was performed on the same set of images in 30 randomly selected cases. Inter-observer analysis showed excellent repeatability and reproducibility. ICC varies between 0.94 and 0.99 for both strain components (GCS and GLS), and 0.93−0.98 for HDF (all *p* < 0.01) (Table 7). 

## 4. Discussion

**Figure 5:**
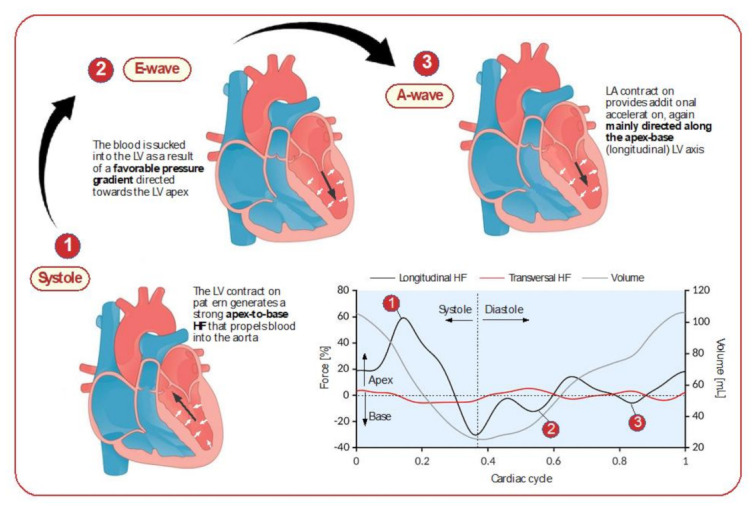
Representation of LV HDFs in the whole cardiac cycle. LV, left ventricular; HDFs, hemodynamic forces.

HDFs introduce novel information about cardiac mechanics. In this regard, looking at the dynamics of blood flow provides an alternative viewpoint to cardiac function. Here, HDFs correspond to the ultimate result of LV contraction–relaxation rhythm and play an important role in the description of cardiac function (Figure 2 and Figure 5).

### 4.1. Previous Studies

IVPGs play an important physiological role [13] as described by several catheterization studies [30,31,32,33]. In fact, the development of IVPGs in the apex-to-based direction ensures efficient ejection of blood into the aorta during systole [34] and at early diastole (suction) represents a key factor for diastolic filling [34,35]. Unfortunately, widespread clinical applications of IVPGs have been limited by the need of invasive measurements. However, with the advent of blood flow imaging technologies, such as echo particle image velocimetry (Echo-PIV) and especially 4D Flow MRI, the IVPGs could be also evaluated non-invasively. The HDFs concept represents a modern approach, a global IVPG measure given by its value averaged over the entire LV volume. Preliminary studies with Echo-PIV demonstrated that HDFs lose their longitudinal alignment by alteration of LV synchrony [18]. The above result have been confirmed by 4D Flow MRI investigations [16,36] demonstrating HDFs as a clear marker of systolic efficiency. However, MRI flow imaging present a certain operational complexity and costs resulting feasible only among limited populations [37]. The availability of HDFs quantification based on STE technology allows more extensive clinical evaluations [38].

Recently, Faganello et al. [39] have applied an extension of the strain echocardiography software package in order to determine HDFs normal limits among a relatively large cohort of healthy subjects (176 subjects; age range: 16–82; 51% women). This study demonstrated that LV systolic longitudinal HDF and LV impulse were higher in men than in women (16.2 ± 5.3 vs. 13.2 ± 3.6; 25.1 ± 7.9 vs. 19.4 ± 5.6 and 20.4 ± 7 vs. 16.6 ± 5.2, *p* < 0.0001, respectively). A weak but statistically significant decline with advancing age was also found for HDFs parameters, following the trend of GLS (all *p* < 0.0001). On the other hand, GCS and LVEF showed an increase with older age (all *p* < 0.0001). 

### 4.2. Uniqueness of the Present Study

To the best of our knowledge, this is the largest study that: (a) comprehensively assessed the full range of HDFs parameters (longitudinal and transversal) in a large cohort of healthy individuals stratified by age and sex; (b) demonstrated systolic HDFs parameters were higher in men than women, except for diastolic HDFs parameters; (c) showed HDFs longitudinal parameters were reduced in older age (GLS and LVEF did not change, while GCS increased with age); (d) revealed age, BSA, pulse pressure, heart rate and GCS were the only independent variables associated with LV longitudinal HDFs. In this regard the interesting paper by Faganello et al. [39] on a healthy population did not report the clinical and echocardiographic correlates of HDF.

Our findings suggest a physiological impact of the aging process more evident on the systolic HDFs parameters (reduced) and GCS (increased) than on GLS and LVEF. The compensatory (to preserve cardiac output) progressive increase of GCS with aging was already described in previous studies [39,40,41]. On the contrary, data relating to GLS and aging in healthy subjects were more controversial [39,42,43]. Our data showed that GLS did not decrease significantly with older age. Minor differences between the study of Faganello et al. [39] and the present study in age population (47 ± 18 vs. 43.4 ± 14 years, respectively) and systolic blood pressure (128 ± 18 vs. 122 ± 12 mmHg, respectively) could explain the differences in GLS related to age. It is likely that the minor degree of increased afterload would be insufficient to influence LV systolic function, as measured by GLS. On the other hand, the significant correlation of HDFs parameters with GCS and pulse pressure may be indicative that HDFs were more influenced by increased arterial stiffening and afterload with advancing age. These effects may increase the susceptibility of aging heart to ventricular dysfunction, suggesting the HDFs as a potential marker of earlier alterations of cardiac LV mechanics. The reference values of HDFs parameters reported in the present study were consistent with previous results, using the same strain-based HDFs technology and software [39]. 

## 5. Limitations

Few study limitations need to be discussed. First, our study was limited to Caucasian healthy subjects. For this reason, the clinical relevance in different races and pathologic states was not assessed. Second, the present study did not validate the accuracy of strain measurements against reference standards such as MRI. The quantitative comparison with previous evaluations with 4D Flow MRI in a relatively smaller number of patients showed larger systolic values (present study: amplitude = 20.8 ± 6.2; Töger et al. 2018 [44]: *n* = 23, amplitude = 15.0 ± 5.0; Arvidsson et al. 2017 [3]: *n* = 39, amplitude = 13.5 ± 6.8) and substantially similar values in diastole (present study: amplitude= 8.1 ± 2.7; Töger et al. 2018 [44]: *n* = 23, amplitude = 10.0 ± 2.5), although details of normalization were not identical. This difference may be partly imputable to imaging technology: 4D flow MRI has a lower temporal resolution that smooths out the sharp systolic acceleration especially during systole, and presents a smoother flow averaged over numerous heartbeats that cancels the sharper local flow peaks. Third, values of GLS and GCS between the different ultrasound vendors were not compared. Inter-vendor variability exists even in full-thickness strain due to the differences in the analytical algorithm. Forth, the apical approach to GCS could be less accurate because the entire circumference was not visible from the apical views. These potential pitfalls were minimized by using a triplane evaluation, commonly used in the evaluation of LV volumes.

## 6. Conclusions

We reported the physiologic range of LV strain and LV-HDFs parameters measured by TTE. Knowledge of age- and gender-specific reference values, for a combination of standard, mechanical and hemodynamic indices may improve the global assessment of the LV function and help to detect sub-clinical stages of LV dysfunction.

## Figures and Tables

**Table 1:** Demographic and clinical characteristics of study population.

Variable	Overall	Women	Men	*p* Value
	(*n* = 269)	(*n* = 146)	(*n* = 123)	
	(Mean ± SD)	(Mean ± SD)	(Mean ± SD)	
Age (years)	43.4 ± 14.0	43.7 ± 13.9	43.1 ± 14.1	0.7
Height (cm)	168 ± 9	162 ± 7	175 ± 7	<0.001
Weight (kg)	70 ± 12	63 ± 9	78 ± 10	<0.001
BMI (kg/m^2^)	24.6 ± 3.1	24.0 ± 3.1	25.4 ± 2.9	<0.001
BSA (m^2^)	1.78 ± 0.19	1.67 ± 0.14	1.91 ± 0.16	<0.001
Systolic BP (mmHg)	122 ± 12	120 ± 13	124 ± 11	<0.001
Diastolic BP (mmHg)	77 ± 8	75 ± 9	78 ± 8	0.007
Mean BP (mmHg)	92 ± 9	90 ± 9	93 ± 8	0.002
Pulse pressure (mmHg)	45 ± 10	44 ± 10	45 ± 9	0.167
HR (b/m)	73 ± 12	74 ± 12	71 ± 12	0.018

BMI, body mass index; BP, blood pressure; BSA, body surface area; HR, heart rate; *p* values indicate sex-related differences.

**Table 2:** Left heart structure and function in study population.

Variable	Overall	Women	Men	*p* Value
	(*n* = 269)	(*n* = 146)	(*n* = 123)	
	(Mean ± SD)	(Mean ± SD)	(Mean ± SD)	
Septal wall thickness in diastole (mm)	8.9 ± 1.4	8.3 ± 1.3	9.6 ± 1.2	<0.001
Inferolateral wall thickness (mm) diastole	8.9 ± 1.4	8.4 ± 1.5	9.6 ± 1.3	<0.001
LV end-diastolic diameter (mm)	45.0 ± 4.0	43.4 ± 3.6	46.9 ± 3.7	<0.001
Proximal ascending aorta (mm)	28.9 ± 3.1	27.8 ± 2.9	30.3 ± 2.8	<0.001
LV mass/BSA (g/m^2^)	85.6 ± 20.2	77.5 ± 17.4	95.4 ± 19.1	<0.001
LA Volume (mL)	32.5 ± 7.1	31.0 ± 6.7	34.4 ± 7.0	<0.001
LV EDV (mL)	99 ± 21	88 ± 15	113 ± 21	<0.001
LV ESV (mL)	37 ± 10	32 ± 8	42 ± 9	<0.001
LV EF (biplane) (%)	64.2 ± 5	63.7 ± 4.4	64.9 ± 5.6	0.049
Mitral Peak E/e’ ratio	5.6 ± 1.5	5.7 ± 1.5	5.5 ± 1.4	0.319
SV (mL)	64 ± 13	60.0 ± 11.6	68.5 ± 12.6	<0.001
CO (L/min)	4.6 ± 1.1	4.4 ± 1.0	4.8 ± 1.1	0.005

BSA, body surface area; CO, cardiac output; E, mitral early inflow velocity; e’, early diastolic mitral annular lateral velocity; EDV, end-diastolic volume; EF, ejection fraction; ESV, end-systolic volume; LA, left atrium; LV, left ventricular; SD, standard deviation; SV, stroke volume indexed; *p* values indicate sex-related differences.

**Table 3:** Structural and functional variables of the right heart and pulmonary circulation in the study population.

Variable	Overall	Women	Men	*p* Value
	(*n* = 269)	(*n* = 146)	(*n* = 123)	
	(Mean ± SD)	(Mean ± SD)	(Mean ± SD)	
RV basal diameter (mm)	34.6 ± 2.9	33.5 ± 2.8	35.8 ± 2.4	<0.001
RV longitudinal diameter (mm)	62.7 ± 6.1	60.6 ± 6.2	65.2 ± 6.1	<0.001
RA Volume (mL)	28.8 ± 7.9	26.0 ± 5.8	32.1 ± 8.8	<0.001
TAPSE (mm)	22.7 ± 2.4	22.7 ± 2.4	22.7 ± 2.4	0.916
S′(cm/s)	13.5 ± 2.2	13.4 ± 2.0	13.7 ± 2.3	0.251
PASP (mmHg)	21.8 ± 4.8	21.5 ± 4.4	22.3 ± 5.3	0.170
RVOT AcT (ms)	136.9 ± 18.3	139.8 ± 18.2	134.5 ± 18.2	0.049

AcT, acceleration time; PASP, pulmonary artery systolic pressure; RA, right atrial; RV, right ventricular; RVOT, right ventricular outflow tract; S’, tissue Doppler–derived tricuspid lateral annular systolic velocity; TAPSE, tricuspid annular plane systolic excursion; *p* values indicate sex-related differences.

**Table 4:** Endocardial strain parameters in study population.

Variable	Overall	Women	Men	*p* Value
	(*n* = 269)	(*n* = 146)	(*n* = 123)	
	Mean ± SD	Mean ± SD	Mean ± SD	
	(95% CI)	(95% CI)	(95% CI)	
GLS, %	−23.1 ± 1.5 (−20.1 to −26.1)	−23.1 ± 1.5 (−20.1 to −26.1)	−23.1 ± 1.5 (−20.1 to −26.1)	1.0
GCS, %	−33.0 ± 3.9 (−25.3 to −40.1)	−33.1 ± 3.9 (−25.4 to −40.8)	−32.8 ± 3.8 (−25.3 to −40.3)	0.4

CI, confidence interval; GCS, global circumferential strain; GLS, global longitudinal strain; *p* values indicate sex-related differences; SD, standard deviation.

**Table 5:** Hemodynamic forces parameters in study population.

Variable	Overall	Women	Men	*p* Value
	(*n* = 269)	(*n* = 146)	(*n* = 123)	
	(Mean ± SD)	(Mean ± SD)	(Mean ± SD)	
	(95% CI)	(95% CI)	(95% CI)	
Longitudinal force, FL				
Whole cycle, %	15.0 ± 4.4 (6.3 to 23.7)	14.6 ± 4.1 (6.5 to 22.7)	15.6 ± 4.7 (6.3 to 24.9)	0.06
Systolic impulse, %	19.8 ± 6.1 (7.8 to 31.8)	18.9 ± 5.6 (7.9 to 29.9)	20.8 ± 6.5 (8.0 to 33.6)	0.009
Systolic, %	20.8 ± 6.2 (8.6 to 33.0)	19.8 ± 5.6 (8.8 to 30.8)	22.0 ± 6.7 (8.8 to 35.2)	0.004
Diastolic, %	8.1 ± 2.7 (2.8 to 13.4)	8.1 ± 2.8 (2.6 to 13.6)	8.1 ± 2.6 (3.0 to 13.2)	0.9
**Transversal force, FT**				
Whole cycle, %	2.4 ± 0.9 (0.6 to 4.2)	2.2 ± 0.8 (0.6 to 3.8)	2.6 ± 1.0 (0.6 to 4.6)	0.004
Systolic, %	2.7 ± 1.0 (0.7 to 4.7)	2.5 ± 0.9 (0.7 to 4.3)	2.9 ± 1.1 (0.7 to 5.1)	<0.001
Diastolic, %	2.1 ± 1.1 (0.0 to 3.3)	2.0 ± 1.0 (0.0 to 4.0)	2.2 ± 1.1 (0.0 to 4.4)	0.1
Alignment angle, °	14.0 ± 3.6 (6.9 to 21.1)	13.5 ± 3.3 (7.0 to 20.0)	14.7 ± 3.3 (8.2 to 21.2)	0.004

*p* values indicate sex-related differences.

**Table 6:** Significant independent relation of left ventricular hemodynamic forces in the overall population with clinical and echocardiographic variables by univariate and multivariate analysis.

Variables Related with Amplitude Heart-Beat Longitudinal	Univariate Analysis			Multivariate Analysis	
	*r*	95% CI	*p* Value	Std Coefficient (β)	*p* Value
Gender	−0.114	−0.230 to 0.006	0.6		
Age (years)	−0.155	−0.270 to −0.036	0.01	−0.232	<0.001
BSA (m^2^)	0.139	0.019 to 0.254	0.02	0.149	0.003
Pulse pressure (mmHg)	0.206	0.089 to 0.319	0.001	0.186	<0.001
Heart rate (bpm)	0.448	0.346 to 0.539	<0.001	0.396	<0.001
LV mass (gr)	0.076	−0.044 to 0.195	0.21		
LA vol (mL)	0.011	−0.109 to 0.131	0.86		
LV EF (%)	0.120	0.000 to 0.237	0.05		
LV SV (mL)	−0.095	−0.213 to 0.025	0.121		
E/e’	−0.119	−0.044 to 0.195	0.052		
GLS	−0.153	−0.268 to −0.034	0.01	−0.056	0.266
GCS	−0.254	−0.390 to −0.170	<0.001	−0.328	<0.001

BSA, body surface area; CI, confidence interval; EF, ejection fraction; GLS, global longitudinal strain; GCS, global circumferential strain; LA, left atrial; LV, left ventricular; SV, stroke volume. The partial correlation test by the Pearson method was used to assess clinically relevant variables with *p* < 0.05, which were then incorporated into the multivariate model assessed by multiple linear regression analysis.

**Table 7:** Intra-and inter-observer variability of GLS, GCS and amplitude heart beat longitudinal HDFs.

Variables	ICC	95% Confidence Interval		*p* Value
		Lower Bound	Upper Bound	
Intra-observer variability				
GLS	0.98	0.96	0.99	<0.01
GCS	0.98	0.95	0.99	<0.01
Amplitude Heart Beat Longitudinal	0.98	0.95	0.99	<0.01
Inter-observer variability				
GLS	0.98	0.94	0.99	<0.01
GCS	0.98	0.94	0.99	<0.01
Amplitude Heart Beat Longitudinal	0.97	0.93	0.98	<0.01

ICC, intraclass correlation coefficient; HDFs, hemodynamic forces; GLS, global longitudinal strain; GCS, global circumferential strain.

## Data Availability

The data that support the findings of this study are available from the corresponding author upon reasonable request.
